# The Role of Age on Beta-Amyloid_1–42_ Plasma Levels in Healthy Subjects

**DOI:** 10.3389/fnagi.2021.698571

**Published:** 2021-08-31

**Authors:** Chiara Zecca, Giuseppe Pasculli, Rosanna Tortelli, Maria Teresa Dell’Abate, Rosa Capozzo, Maria Rosaria Barulli, Roberta Barone, Miriam Accogli, Serena Arima, Alessio Pollice, Vincenzo Brescia, Giancarlo Logroscino

**Affiliations:** ^1^Center for Neurodegenerative Diseases and the Aging Brain, Department of Clinical Research in Neurology of the University of Bari “Aldo Moro” at “Pia Fondazione Card G. Panico” Hospital Tricase, Lecce, Italy; ^2^Department of Computer, Control, and Management Engineering Antonio Ruberti (DIAG), La Sapienza University, Rome, Italy; ^3^Department of History, Society and Human Studies, University of Salento, Lecce, Italy; ^4^Department of Economics and Finance, University of Bari “Aldo Moro”, Bari, Italy; ^5^Unit of Laboratory Medicine, “Pia Fondazione Card. G. Panico” Hospital Tricase, Lecce, Italy; ^6^Department of Basic Medicine Sciences, Neuroscience, and Sense Organs, University of Bari “Aldo Moro”, Bari, Italy

**Keywords:** beta amyloid, Alzheimer’s disease, biomarker, age, plasma

## Abstract

Beta-amyloid (Aβ) plaques have been observed in the brain of healthy elderlies with frequencies strongly influenced by age. The aim of the study is to evaluate the role of age and other biochemical and hematological parameters on Aβ_1–42_ plasma levels in cognitively and neurologically normal individuals. Two-hundred and seventy-five normal subjects stratified by age groups (<35 years, 35–65 years, and >65 years) were included in the study. Aβ_1–42_ plasma levels significantly correlated with age (*r_s_* = 0.27; *p* < 0.0001) in the whole sample, inversely correlated with age in the first age group (*r_s_* = −0.25, *p* = 0.01), positively correlated in the second group (*r_s_* = 0.22, *p* = 0.03), while there was no significant correlation in the older group (*r_s_* = 0.02, *p* = 0.86). Both age (*β*-estimate = 0.08; *p* < 0.001) and cholesterol (*β*-estimate = 0.03; *p* = 0.009) were significantly associated with Aβ_1–42_ plasma level in multivariable analysis. However, only the association with age survived *post hoc* adjustment for multiple comparisons. The different effects of age on the Aβ level across age groups should be explored in further studies to better understand the age-dependent variability. This could better define the value of plasma Aβ as a biomarker of the Alzheimer neuropathology.

## Introduction

Alzheimer’s disease (AD) is a progressive neurodegenerative disorder developed as a result of multiple factors rather than a single cause (Alzheimer’s Association, 2019). Age is one of the main risk factors, with the vast majority of people with Alzheimer’s dementia being age 65 or older. The percentage of people with AD increases dramatically with age: 3% of people age 65–74, 17% of people age 75 to 84, and 32% of people age 85 or older (Hebert et al., 2013; Alzheimer’s Association, 2019).

Beta-amyloid (Aβ) deposition is part of the histopathological definition of AD and *in vivo* biomarkers of this process have been included in 2011 by the National Institute on Aging and Alzheimer’s Association (NIA-AA) in the new diagnostic criteria for all the preclinical and clinical stages of the disease (Sperling et al., 2011; Jack et al., 2012). In addition to NIA-AA, the International Work Group (IWG) in 2014 has established diagnostic guidelines for AD that incorporate imaging and cerebrospinal fluid (CSF) biomarkers (Dubois et al., 2014). Based on the IWG criteria, the diagnosis of AD requires the presence of cognitive symptoms plus biomarker evidence of AD pathophysiologic processes. The central role of these biomarkers has been confirmed in the 2018 NIA-AA research framework that biologically defined AD throughout the entire course of the disease (Jack et al., 2018).

Aβ is supposed to trigger a series of biochemical processes that determine tau-deposition, neuronal disruption, neuronal death, and finally clinically manifest AD (“amyloid cascade” hypothesis; Hardy and Allsop, 1991).

Sequential cleavage of Aβ precursor protein (AβPP) by β— and γ—secretases results in the production of multiple Aβ species, the main forms containing 40 (Aβ_1–40_) or 42 amino acids (Aβ_1–42_; Selkoe, 2001). Studies have shown that Aβ exists in a dynamic equilibrium of soluble monomeric, oligomeric, protofibrillar, and fibrillar forms (Dahlgren et al., 2002), reflecting a balance between their production and removal from the brain. Aβ_1–40_ is more soluble, less prone to parenchymal deposition, but more likely to accumulate in the walls of cerebrocortical and leptomeningeal blood vessels, whereas Aβ_1–42_ is relatively insoluble in the interstitial fluid and prone to parenchymal deposition (Iwatsubo et al., 1994; Gravina et al., 1995).

Excessive accumulation of Aβ_1–42_ increases the aggregation of Aβ to form oligomers and fibrils (Pauwels et al., 2012).

Although Aβ plaques are supposed to trigger the AD pathophysiologic process, they are also commonly observed in the brains of clinically normal (CN) older individuals, but the age at which Aβ plaque deposition begins is unknown (Mormino, 2014). This feature has consistently been observed in postmortem studies and has been replicated in amyloid imaging studies (Jack et al., 2014). These studies reveal a low proportion of Aβ deposition in CN individuals younger than 60 years, followed by a linear increase in the proportion of Aβ in CN subjects after the age of 60 (~30% of CN are Aβ positive at age 75; Mormino, 2014).

Many investigators have examined the association between Aβ and brain changes in CN to determine whether Aβ accumulation in normal subjects could signal AD preclinical state, but often with inconsistent results (Hardy and Selkoe, 2002; Jack et al., 2013; Villemagne et al., 2013; Fandos et al., 2017). However, little is known about the general demographic, clinical, biochemical, and hematological factors impacting plasma Aβ levels. Studies have investigated different biochemical blood parameters such as creatinine (Arvanitakis et al., 2002; Irizarry et al., 2005; Luchsinger et al., 2007; Metti et al., 2013; Rajagopalan et al., 2013), total cholesterol, High-density Lipoprotein (HDL) cholesterol, bilirubin, platelets (Toledo et al., 2011), and Thyroid Stimulating Hormone (TSH; Tan et al., 2008; Choi et al., 2017) with uncertain and variable results.

The goal of this study is to evaluate plasma levels of Aβ_1–42_ in a sample of cognitively and neurologically normal individuals with a wide age-range 19–89 years, in order to study the variability and trends in Aβ_1–42_ plasma levels by age, and whether plasma levels are influenced by biochemical and hematological blood parameters.

## Materials and Methods

### Study Population

The sampling strategy included the enrollment of a broad age-range population. Three age-groups (<35 years; 35–65 years; >65 years) were then considered for statistical purposes and the number of enrolled subjects was equally distributed among groups.

Younger (<35) and middle-aged subjects (35–65 years) were enrolled from the blood-donor service of the donation site located in the “Azienda Ospedaliera Card. G. Panico,” Tricase Lecce (Panico cohort); all donors were informed of the possibility to join the study within the normal donation process. Past medical history (presence of any identified neurological or medical condition) was investigated through a structured questionnaire administered before the blood draw.

The older participants (>65 years) were enrolled from the GreatAGE Study, a population-based study on neurological and psychiatric age-related diseases with a focus on nutrition and age-related hearing loss as predictors of late-life cognitive decline and depression, conducted in the area of Castellana Grotte, Southern Italy (GreatAGE cohort; Lozupone et al., 2018).

A detailed description of the methods used for the clinical assessment of the study population has been published elsewhere (Zecca et al., 2018).

Exclusion criteria considered for this study were: (1) the presence of signs/symptoms of any neurological or psychiatric diseases (or previous diagnosis) documented at the time of enrollment; (2) pharmacological therapy at the time of enrollment; (3) any illness in the previous 3 months that required medical intervention; (4) history of chronic liver, kidney or thyroid diseases; and (5) current drug or alcohol addiction.

The present study was approved by the Ethics Committee of ASL Lecce and by the Institutional Review Board of the “National Institute of Gastroenterology “S. De Bellis”. All participants gave written informed consent.

### Blood Sampling, Biochemical Determinations, and Aβ_1–42_ Measurements

Venous blood was drawn by venipuncture in the morning after an overnight fast. Plasma samples were collected in EDTA vacutainers, which were immediately centrifuged for 5 min at 3,000 *g* at room temperature. Blood samples were routinely processed for hematologic and biochemical measurements, according to routine clinical standards. Plasma samples were aliquoted into polypropylene tubes and stored at −80°C until biochemical analyses (without being thawed and re-frozen) for blood amyloid testing. Samples were thawed at room temperature before analysis. Only plasma samples free from hemoglobin, bilirubin, and triglycerides, which could interfere with the analytical methods, were considered eligible for analysis.

Quantification of Aβ_1–42_ in plasma was performed using a specific ELISA kit (Innotest β-amyloid_1–42_, Innogenetics, Belgium), according to the manufacturer’s instructions. The assay involved the use of a high sensitivity conjugate for the detection of the protein in plasma samples. Briefly, immunocoated plates were incubated with 100 μl of sample or calibrator for 3 h at room temperature on an orbital shaker. After several wash steps, a biotinylated antibody was added to the plates and incubated for 1 h at room temperature. This antibody was then detected by a peroxidase-labeled streptavidin. After the addition of substrate solution, positive samples developed a blue color. The reaction was stopped by the addition of sulfuric acid and the absorbance was then measured at 450 nm. All samples were analyzed in duplicate for each test run. Plasma Aβ_1–42_ levels were presented as pg/ml. A reference interval of 8.12–29.00 pg/ml for Aβ_1–42_ plasma levels was considered (Zecca et al., 2018).

The following biochemical parameters were examined: glucose, urea, creatinine, aspartate aminotransferase (AST), alanine aminotransferase (ALT), gamma glutamyl transpeptidase (gamma-GT), total bilirubin, total cholesterol, triglycerides, high-density lipoprotein (HDL) cholesterol, phosphorus, calcium, thyroid-stimulating hormone (TSH), full blood cell count (white cells with differentials, red cells, and platelets), hemoglobin (Hb) and erythrocyte sedimentation rate (ESR).

### Statistical Analysis

Summary results are presented as mean ± standard deviation (SD) for normal continuous variables, the median-interquartile range for the non-normally distributed ones, and as absolute frequencies (with percentage frequencies in brackets) for categorical variables. The Shapiro-Wilk test was used to assess the normal distribution of the continuous variable residuals in a linear model against age categories. Continuous and categorical variables were compared across age subgroups using the Kruskal-Wallis, ANOVA, and the Pearson Chi-Square tests, respectively for continuous (non-normally and normally distributed) and categorical covariates. Spearman correlation corrected for multiple tests (Benjamini-Hochberg procedure) was used to test for the proportion of variance in the ranks shared between continuous covariates and Aβ_1–42_ levels.

Unpaired *t*-test was used to evaluate differences of Aβ_1–42_ levels between males and females stratifying by the three age groups. One-way analysis of means not assuming equal variances (Welch’s F Test) was used to test differences in Aβ_1–42_ levels between males and females in the overall (not age-stratified) dataset. The latter test was also used to compare Aβ_1–42_ levels between different age groups (as a result of significative Levene’s test for homogeneity Aβ levels variance across age groups, *p* < 0.01). All *p*-values obtained by age groups pairwise comparison were adjusted for family-wise error with the Bonferroni method. Non-parametric (Kruskal-Wallis) or parametric (Linear ANOVA) methods were used according to linear regression residual distributions to compare levels of other covariates between the three age groups. Piecewise cubic polynomial 97.5% confidence intervals (Figure 2) were obtained after *n* = 1,000 bootstraps data points replicates.

**Figure 1:**
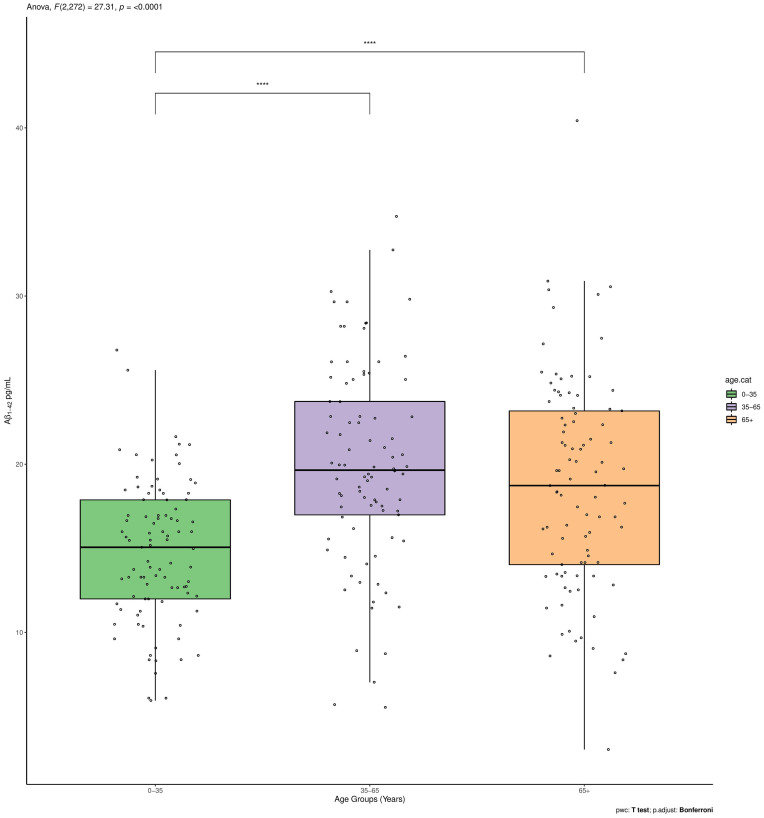
Aβ_1–42_ plasma levels between the three age groups (<35, 35–65, >65). ****p < 0.001.

**Figure 2:**
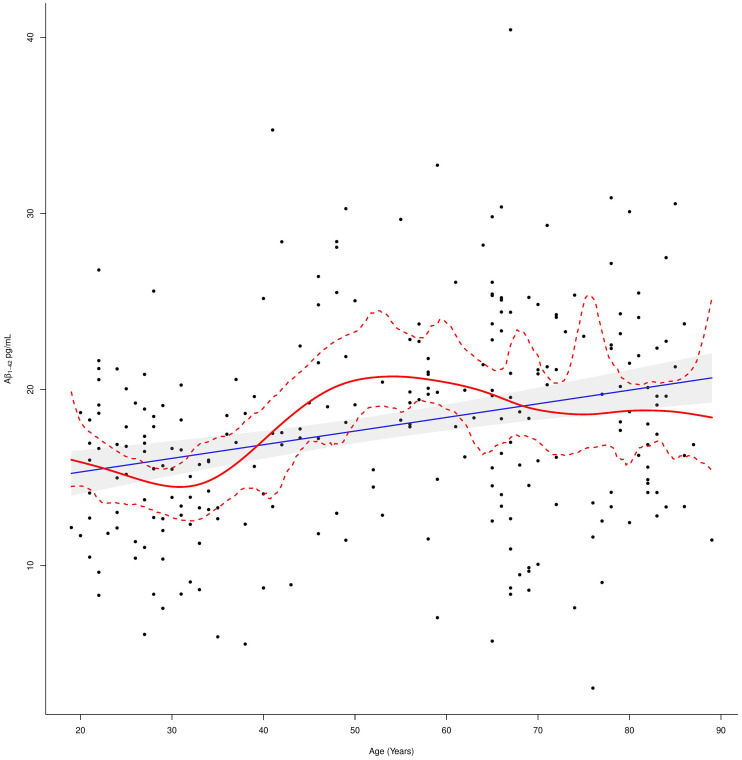
Plasma Aβ_1–42_ levels in relation to age. The linear regression fit line (blue line) and the cubic smoothing spline (red line) were added. The 97.5% confidence limits around the main spline estimate are based on 1,000 bootstrap re-samplings of the data points in the scatterplot.

Finally, stepwise (both direction selection) multivariable linear regression models were run with Aβ_1–42_ as a dependent variable to determine which factors predicted Aβ_1–42_ protein levels. We used a stepwise selection process with significance levels *α* = 0.05 for covariate deletions and *α* = 0.20 for covariate additions, in order to determine the final multivariable models. All analyses were performed using R version 3.6.2 running under Windows 10 x64 (build 18362).

## Results

Two hundred and seventy-five cognitively and neurologically normal subjects were enrolled (120 women and 155 men; age range, 19–89 years; mean age ± SD, 51.61 ± 21.08 years). According to the sampling strategy, the number of subjects was equally distributed among age groups, with 93 subjects in the younger age group (<35 years), 89 subjects in the middle-age group (35–65 years), and 93 subjects in the older age-group (>65 years).

Descriptive statistics of the demographic characteristics, and of the biochemical and hematological indices of the whole sample as long as of the three subgroups are shown in Table 1.

**Table 1:** Demographic characteristics and baseline biochemical and hematological indices.

	<35	35–65	>65	**Total**	*p* value
N	93	89	93	275
Age (years)	27.17 (4.53)	52.30 (9.19)	75.40 (6.78)	51.61 (21.08)	<0.001^1^
Sex					<0.001^2^
F	57 (61.29%)	38 (42.70%)	25 (26.88%)	120 (43.64%)	
M	36 (38.71%)	51 (57.30%)	68 (73.12%)	155 (56.36%)
Aβ_1–42_ (pg/ml)	14.76 (4.16)	19.96 (5.96)	18.67 (6.36)	17.76 (5.97)	<0.001^3^
AST (U/L)	23.41 (9.46)	25.82 (8.73)	23.46 (12.56)	24.21 (10.43)	0.002^1^
ALT (U/L)	24.89 (6.09)	26.03 (9.09)	22.44 (13.61)	24.43 (10.17)	<0.001^1^
GGT (U/L)	25.49 (8.20)	26.17 (11.88)	22.30 (23.65)	24.63 (16.08)	<0.001^1^
Bilirubin (μmol/L)	0.76 (0.43)	0.75 (0.34)	0.64 (0.31)	0.72 (0.37)	0.047^1^
Cholesterol (mmol/L)	179.06 (26.53)	180.56 (29.59)	179.20 (35.86)	179.60 (30.82)	0.937^4^
HDL (mmol/L)	49.51 (10.49)	42.88 (10.16)	48.41 (13.11)	46.99 (11.67)	<0.0011
Triglycerides (mmol/L)	110.97 (45.86)	108.46 (33.32)	97.96 (50.95)	105.78 (44.32)	0.005^1^
Creatinine (μmol/L)	0.69 (0.26)	0.70 (0.24)	0.96 (0.32)	0.79 (0.30)	<0.001^1^
Glucose (mmol/L)	81.62 (10.94)	85.34 (15.62)	101.38 (20.89)	89.51 (18.43)	<0.001^1^
Total Protein (g/L)	7.29 (0.52)	7.31 (0.58)	6.88 (0.48)	7.16 (0.56)	<0.001^1^
TSH (mIU/L)	1.58 (0.73)	1.38 (0.66)	1.72 (0.95)	1.56 (0.80)	
Urea (mmol/L)	27.80 (6.41)	28.00 (9.67)	42.18 (10.66)	32.73 (11.31)	<0.001^1^
WBC (10E9/L)	6.45 (1.66)	6.21 (1.54)	5.82 (1.54)	6.16 (1.60)	
Erythrocytes (10E12/L)	4.89 (0.55)	4.85 (0.56)	4.85 (0.56)	4.86 (0.56)	0.898^1^
Platelets (10E9/L)	242.14 (54.80)	244.35 (58.75)	205.81 (49.05)	230.57 (56.92)	<0.001^1^
Hb (g/L)	13.58 (1.53)	13.69 (1.55)	13.78 (1.61)	13.68 (1.56)	0.674^4^

*AST, aspartate aminotransferase; ALT, alanine aminotransferase; GGT, gamma glutamyl transpeptidase; HDL-cholesterol, high-density lipoprotein cholesterol; TSH, thyroid-stimulating hormone; WBC, white blood cells; Hb, hemoglobin. The results are reported as mean ± standard deviation (SD) Comparisons by age groups. ^1^Kruskal-Wallis rank sum test. ^2^Pearson’s Chi-squared test. ^3^One-way analysis of means (not assuming equal variances). ^4^Linear Model ANOVA*.

Aβ_1–42_ plasma levels fitted a normal distribution in the whole study population (*p* = 0.06) as well as in the age subgroups (*p* = 0.59 for <35 age subgroup; *p* = 0.73 for 35–65 age subgroup; *p* = 0.38 for >65 age subgroup). The mean value (± SD) of the biomarker was 17.76 (±5.97) pg/ml in the whole group, 14.76 (±4.16) in the first age group, 19.96 (±5.96) in the second age group, and 18.67 (±6.36) in the third age group.

The Aβ_1–42_ plasma levels significantly differed between the three age groups (*p* < 0.001). Such significant overall difference between age groups was also confirmed for two of the subgroups with pairwise comparisons using *t*-tests with non-pooled SD as a *post hoc* analysis (<35 vs. 35–65 *p* < 0.0001; <35 vs. >65 *p* < 0.0001; 35–65 vs. >65 *p* = 0.36; Figure 1).

Furthermore, statistically significant differences were found also for most of the biochemical and hematological parameters between age subgroups (Table 1). No differences were found for erythrocytes (Kruskal-Wallis test, *p* = 0.90), Hb (Linear Model Anova, *p* = 0.65) among the three age groups.

The correlations between Aβ_1–42_ plasma levels and demographic and clinical parameters in the whole sample and in the three age strata are described in Table 2.

**Table 2:** Correlations between Aβ_1–42_ measurements and clinical parameters observed in this study.

Variable		Overall			1st Group			2nd Group			3rd Group		
		Aβ_1–42_/r_s_	*p*-value	adj.p	Aβ_1–42_/r_s_	*p*-value	adj.p	Aβ_1–42_/r_s_	*p*-value	adj.p	Aβ_1–42_/r_s_	*p*-value	adj.p
Sex	M	17.89 ± 6.45	0.67		14.55 ± 5.00	0.71		21.09 ± 6.06	0.20		18.85 ± 6.88	0.94
	F	17.59 ± 5.31			14.89 ± 3.57			18.95 ± 5.76			18.31 ± 4.77
Age		0.27	**<0.0001**	**<0.0001**	−0.25	**0.01**	0.56	0.22	**0.03**	0.52	0.02	0.86	0.95
ALT		−0.06	0.31	0.66	0.03	0.76	0.78	0.08	0.47	0.98	−0.17	0.09	0.58
AST		−0.04	0.48	0.50	−0.16	0.12	0.97	−0.01	0.93	0.87	−0.12	0.25	0.37
GGT		−0.01	0.86	0.91	−0.01	0.94	0.99	0.2	0.06	0.56	0.02	0.85	0.95
Bilirubin		−0.01	0.84	0.90	0.07	0.51	0.96	−0.05	0.65	0.95	−0.06	0.55	0.82
Cholesterol		0.12	**0.04**	0.13	−0.06	0.56	0.97	0.2	0.06	0.56	0.18	0.08	0.34
HDL		−0.06	0.31	0.41	0.12	0.25	0.97	−0.05	0.62	0.95	0.06	0.56	0.26
Triglycerides		0.07	0.22	0.50	−0.04	0.67	0.93	0.03	0.80	0.95	0.21	**0.04**	0.82
Creatinine		0.08	0.17	0.35	0.02	0.82	0.97	−0.07	0.49	0.87	0.13	0.22	0.55
Glucose		0.09	0.14	0.37	−0.11	0.27	0.93	−0.02	0.88	0.98	0.08	0.46	0.78
Total proteins		−0.11	**0.05**	0.16	0.1	0.36	0.93	−0.15	0.15	0.78	−0.2	**0.05**	0.29
TSH		0.06	0.37	0.50	−0.09	0.38	0.93	0.1	0.37	0.87	0.22	0.04	0.24
Urea		0.09	0.13	0.31	−0.01	0.93	0.99	0.04	0.74	0.95	0.03	0.8	0.95
RBC		−0.06	0.28	0.34	−0.13	0.22	0.97	0.09	0.42	0.99	−0.14	0.18	0.51
WBC		−0.09	0.15	0.48	0.05	0.67	0.93	0	0.99	0.87	−0.15	0.16	0.53
Platelets		−0.04	0.56	0.71	−0.1	0.34	0.93	0.08	0.44	0.87	−0.01	0.93	0.99
Hb		0.02	0.70	0.83	−0.03	0.78	0.97	0.04	0.74	0.95	0.01	0.93	0.99

*P-values refer to the Spearman correlation test (adj.p for Benjamini-Hochberg procedure for multiple testing) for continuous variables and to the *t*-test (Welch test for unequal variances for the overall groups) for categorical variable*. *Bold values denote statistical significance at the *p* ≤ 0.05 level*.

There were no differences in the mean plasma levels of Aβ_1–42_ between women (mean = 17.59, SD = 5.31) and men (mean = 17.89, SD = 6.45; *p* = 0.67) in the whole age group nor in any of the three age-related strata (*p* = 0.71 for <35 age group, *p* = 0.20 for 35–65 age group, *p* = 0.94 for >65 age group).

Aβ_1–42_ plasma levels correlated significantly with age (*r_s_* = 0.27; *p* < 0.0001), in the whole group.

These evidences suggest that Aβ_1–42_ levels in cognitively normal individuals overall increased with age although this trend was not linear as demonstrated by fitting a cubic smoothing spline to our data (Figure 2).

In fact, Aβ_1–42_ plasma levels decreased in the first age group and then steadily increased in the second age group, to end up with another slight decrease up to a steady level in the third age group. In order to detect these age-dependent fluctuations of the protein levels and to study the effect of possible confounders, a subgroup correlation analysis was conducted. Spearman correlations in the three age groups, confirmed that Aβ_1–42_ plasma levels negatively correlated with age in the first age group (*r_s_* = −0.25; *p* = 0.01), positively correlated in the middle-age group (*r_s_* = 0.22; *p* = 0.03), while no significant correlation was detected in the third age group (*r_s_* = 0.02; *p* = 0.86,). After correcting for multiple tests, the correlation with age was confirmed in the whole group (*p*.adj = < 0.0001) and not confirmed in the first and middle-age group (*p*.adj = 0.56 and *p*.adj = 0.52, respectively) although such values were found significant in the crude analysis (Table 2).

Finally, for the whole sample and for each subgroup, a bidirectional stepwise multivariable linear regression model was fitted. Results are shown in Table 3.

**Table 3:** Stepwise multivariable (both directions selection) linear regression analysis exploring factors correlated with Aβ1–42.

	Overall			Group1 (<35)			Group2 (35–65)			Group3 (>65)		
Predictors	*β*-Estimate	96% CI	*p*-value	*β*-Estimate	96% CI	*p*-value	*β*-Estimate	96% CI	*p*-value	*β*-Estimate	96% CI	*p*-value
(Intercept)	8.50	4.15–12.84	**<0.001**	25.35	17.56–33.15	**<0.001**	9.37	1.43–17.30	**0.021**	35.40	16.03–54.76	**<0.001**
Age	0.08	0.04–0.11	**<0.001**	−0.24	−0.42 to −0.05	**0.011**	0.14	0.01–0.28	**0.038**			
Total Cholesterol	0.03	0.01–0.05	**0.009**							0.04	0.00–0.07	**0.042**
Creatinine				2.75	−0.43–5.93	0.089						
Glucose				−0.07	−0.15–0.00	0.055						
TSH							2.30	0.43–4.17	**0.016**			
Total Proteins										−3.40	−6.03 to −0.76	**0.012**
Observations	274			93			89			92
R^2^ / R^2^ adjusted	0.096/0.089			0.123/0.093			0.095/0.074			0.111/0.091

*Bold values denote statistical significance at the *p* ≤ 0.05 level*.

In the model for the whole sample, a significant regression equation was found (*p* < 0.01), with an R^2^ of 0.1. Both age (*β*-estimate = 0.08; *p* < 0.001) and cholesterol (*β*-estimate = 0.03; *p* = 0.009) were significantly associated with biomarker levels although the latter, differently from the more solid age findings, might be an artifact product of the stepwise regression analysis. Age was confirmed to be significantly associated with biomarker levels also in the first (*β*-estimate = −0.24; *p* = 0.011) and second (*β*-estimate = 0.14; *p* = 0.038) age groups. No significant associations were found between age and Aβ_1–42_ levels in the stepwise multivariable linear regression model for the third age group (age covariate was omitted after the stepwise selection process for this age group). In addition, Aβ_1–42_ levels were significantly associated with TSH (*β*-estimate = 2.30; *p* = 0.016) in the 35–65 age group and with total cholesterol and total proteins (*β*-estimate = 0.04, *p* = 0.042; *β*-estimate = −3.40, *p* = 0.012) in the last age group (>65 age). Assumption tests for statistical modeling were undertaken on the overall final model and were reported in Supplementary Material.

## Discussion

In the present study, we evaluated the Aβ_1–42_ plasma levels in cognitively normal individuals with a wide range of age (19–89 years) in order to study if and how they are influenced by age.

We found an overall positive correlation between Aβ_1–42_ plasma levels and age. Our results suggested that Aβ_1–42_ levels, in cognitively normal individuals, increase with age although this trend is not linear: we observed a reduction of Aβ_1–42_ plasma levels in the younger cases, followed by an increase in the adult age class and subsequent stabilization over 65 years.

This is in line with previous studies (Fukumoto et al., 2003; Toledo et al., 2013; Miners et al., 2014) that have highlighted the association of age with differential changes in protein levels, although, a study, dividing 391 subjects by 10-years intervals starting from age 20–29 years, reported a negative correlation between age and Aβ_1–42_ plasma concentration, and no age-group differences (Lue et al., 2019). Probably the differences with this study may be due to the different analytical methods.

Our findings on protein levels in subjects over 65 years were comparable with those found by de Wolf et al. (2020) in a population-based cohort study including subjects of the same age group. The authors investigated whether levels of Aβ_1–42_ plasma of non-demented subjects >60 years of age were associated with AD dementia, arguing that lower Aβ_1–42_ plasma levels are significantly associated with incident AD dementia. In line with our results, there was no correlation between Aβ_1–42_ and age in the 65-year-old subjects.

Age represents an important factor to consider when evaluating the accuracy of a diagnostic test and is an additive information to the biochemical data. A recent study (West et al., 2021) based on plasma Aβ42/40 ratio has in fact highlighted how plasma Aβ42/40 concentration ratio determined using the Mass Spectrometry assay can accurately identify brain amyloid status, and that including additional risk factors for amyloid pathology in the model, as age or ApoE4 status, improved the model accuracy. Given the central role for Aβ in AD and the need for biomarkers that can be used as reliable diagnostic tools, many studies have examined the temporal dynamics of Aβ in biological samples (Huang et al., 2012a, b; Moghekar et al., 2012) and it has also been largely investigated whether Aβ concentration can be influenced by age (Mayeux et al., 2003; Song et al., 2011; Toledo et al., 2011), by testing the longitudinal changes in plasma Aβ levels in cognitively stable individuals vs. those who develop AD dementia and the concentration across time at different stages of the disease. In cognitively stable individuals, plasma Aβ levels increase slightly with age. It has been hypothesized that the age-related increase of Aβ species in plasma may reflect in the periphery the increased Aβ production or decreased Aβ clearance in the brain leading to increased Aβ deposition and AD with aging (Fukumoto et al., 2003).

In subjects who eventually develop clinical AD, protein levels are elevated in the pre-dementia stage, reach a peak and then diminish prior to the development of clinical AD symptoms (Song et al., 2011).

The decrease of plasma amyloid level during the Alzheimer’s process could be explained by the decrease of Aβ clearance from the brain to the peripheral fluid (blood) because of alteration of blood-brain barrier permeability, glymphatic system, or vascular or microglial activation troubles (Ramanathan et al., 2015).

What emerged in our study found agreement with imaging (Rodrigue et al., 2012; Jack et al., 2014) and postmortem (Price et al., 2009) studies, that showed a steady increase in plaque deposition across the age span of 26–95 years, with a slowing of the age-related increase in plaques at the older end of the lifespan. Savva and colleagues (Savva et al., 2009) conducted neuropathological examinations on the brains of about 500 older individuals (aged 69–103 years) for whom the dementia status was known from assessments conducted, on average, 1.5 years before death. The density of Alzheimer-type pathology (neurofibrillary tangles and neuritic plaques) and the severity of other pathologies (atrophy, cerebrovascular disease, and Lewy bodies) were evaluated in the cerebral cortex and hippocampus of these brains, and five age groups for analysis (≤80, 80–84, 85–89, 90–94 and ≥95 years of age) were considered. The study highlighted that the prevalence of Alzheimer-type pathology progressively increased with age in both brain regions of individuals without dementia. In contrast, in the brains of people with dementia, the prevalence of such pathology remained constant or decreased with increasing age. The findings from the study indicated that in the younger old (<80 years of age) the presence of moderate or severe Alzheimer-type pathology was strongly associated with dementia, but the strength of the associations progressively declined with age and was at its weakest in the oldest old (≥95 years of age).

Aβ peptides are generated outside of the central nervous system in appreciable quantities by the skeletal muscle, platelets, and vascular walls (Roher et al., 2009). As well as, amyloid precursor protein (APP), the only member of the family encoding Aβ peptides, is expressed in endothelial cells of cerebral and peripheral arteries, with physiological and pathological implications, from atherosclerosis to cerebral amyloid angiopathy (d’Uscio et al., 2017). It has been demonstrated that APP and Aβ are increased in plasma of patients with coronary heart disease (Stamatelopoulos et al., 2015).

Moreover, recent studies have identified Aβ as an antimicrobial peptide (AMP), and suggest Aβ deposition may be a protective innate immune response to infection (Soscia et al., 2010; Kumar et al., 2016; Eimer et al., 2018). Such peptides act both by directly inactivating pathogens, and also by modulating responses of innate immune cells, including phagocytes. These antimicrobial properties have been attributed to Aβ oligomers that form fibrils in the presence of bacterial surface epitopes (Voth et al., 2020). Amyloids, particularly Aβ42, polymerizes into fibrils upon contact with bacterial surface epitopes and actively agglutinate bacteria prior to bactericidal activity. It has been shown that pathogens responsible for nosocomial pneumonia, including *Pseudomonas aeruginosa*, *Klebsiella pneumonia*, and *Staphylococcus aureus*, elicit lung endothelial production and release of amyloids (Voth et al., 2020). So, all these potential sources of Aβ should be taken into consideration when evaluating peripheral levels of the protein.

In order to assess whether other factors may affect Aβ protein, the relationships between Aβ_1–42_ plasma levels and biochemical/hematological blood parameters have also been explored in the present study.

In line with previous studies (Toledo et al., 2011; Metti et al., 2013; Ruiz et al., 2013), we found an association between Aβ_1–42_ and cholesterol, total protein, creatinine, HDL, and platelets.

According to these studies, the age and the above mentioned parameters were independent predictors for Aβ_1–40_ and Aβ_1–42_ and explained 12.1% and 12.9% of the variability of their respective concentrations, underscoring the importance of using multivariable models that adjust for possible confounders (Toledo et al., 2011).

In our study, both directions stepwise multivariable linear regression models were examined for Aβ_1–42_ in the whole sample and in each subgroup to determine which factors independently predict biomarker levels. In the model for the whole sample, biomarker levels were significantly predicted by age and total cholesterol. Our results agree with a population-based study of subjects over 75 that found higher total cholesterol and higher LDL cholesterol predicted plasma Aβ_1–42_ levels (Blasko et al., 2011). The proposed mechanism by which cholesterol might accelerate the production of Aβ is by shifting Aβ precursor protein (AβPP) metabolism from forming alpha to beta cleavage products (Blasko et al., 2011). Also in the study of Toledo and coworkers, Aβ_1–40_ and Aβ_1–42_ plasma levels were mainly predicted by creatinine, total protein, and total cholesterol (Toledo et al., 2011); however, another study did not support these findings, showing different significant predictors of plasma Aβ_1–40_ and Aβ_1–42_ (history of diabetes, HDL cholesterol; Metti et al., 2013).

The stepwise linear regression analysis stratified by age subgroups detected various relations inside the three groups; age was significantly associated with plasma protein concentrations in the first group (<35 years), age and TSH in the second group (35-65 years), and total cholesterol and total proteins in the third group (>65 years). A study has shown a positive correlation between TSH and triglycerides and Aβ_1–42_ plasma levels in cognitively intact subjects over 65 (Tan et al., 2008). Several *in vitro* and *in vivo* studies have shown that thyroid hormone regulates the gene expression of amyloid precursor protein (APP), increasing APP expression and consequently, Aβ peptide and Aβ levels (O’Barr et al., 2006).

The presence of conflicting results between studies assessing plasma Aβ peptides can be explained, in part, by the high variability in the methods for Aβ_1–42_ measurements, not yet standardized; different technologies have been used to measure plasma Aβ (ELISA, Luminex, or Simoa technology), with different diagnostic performance. But also, the study designs or the different ages of subjects enrolled can affect the comparison of results.

Some limitations need to be considered in this study. Firstly, we did not have any CSF and positron emission tomography (PET) measurements of Aβ_1–42_ and thus it was not possible to correlate plasma Aβ_1–42_ to measurements or burden of Aβ_1–42_ in CSF to corroborate our results. Secondly, the value of plasma Aβ42/40 ratio as surrogate biomarkers of cortical Aβ deposition was not available. The ratio appears to be a better predictor of the presence of brain amyloid than just the plasma Aβ_1–42_ concentration. Moreover, the ratio, instead of single peptide measurements, could also attenuate possible bias in single Aβ peptide levels due to pre-analytical and analytical variables. Thirdly, the use of a classic ELISA method for plasma quantification of protein levels. From a molecular biology point of view, a more sensitive methodology would have been optimal for better detection and accuracy in measuring blood amyloid protein. The recent introduction of new ultrasensitive assays, such as a single-molecule array, allows detection at a single molecule level, significantly improving analytical sensitivity, and their use in research is strongly recommended. Sensitive measurement of plasma Aβ levels in a large patient group is required to clarify the clinical, demographic, and genetic factors that influence plasma Aβ levels, and as a prerequisite for proposing plasma Aβ as a biomarker for diagnosis, progression, and treatment effects.

We point out that the data we analyzed came from a retrospective study that, as we know from literature (Cowie et al., 2017), are important tools in medical research. Nonetheless, we are conscious that such kinds of studies have several limitations owing to their design such as selection or recall biases (Talari and Goyal, 2020). However, the main strengths of this study are the large sample size and the wide age range of healthy individuals, but also the assessment of normality, especially for the older participants (>65 years), with an extensive geriatric and neurological examination and standardized cognitive tests. Moreover, all the Aβ measurements were undertaken in a single-center laboratory, eliminating inter-center variability.

To conclude, after age 65 Aβ_1–42_ does not increase in normal subjects, and in the whole cohort age and Aβ_1–42_ are strongly associated.

Our findings open new insights to better understand the effect of age on plasma Aβ peptides.The possible relationship of Aβ with age should be further explored in larger longitudinal studies, especially exploring the oldest age, and comparing data from wet biomarkers with imaging and neuropsychological data. Furthermore, our results may be important to better define the diagnostic value of plasma Aβ in studies of age-related diseases.

## Data Availability Statement

The raw data supporting the conclusions of this article will be made available by the authors, without undue reservation.

## Ethics Statement

The studies involving human participants were reviewed and approved by Ethic Committee of ASL Lecce. The patients/participants provided their written informed consent to participate in this study.

## Author Contributions

Manuscript design and writing: CZ, RT, MTD, and GL. Data analysis: GP, AP, and SA. Data collection: CZ, RT, MTD, RC, MB, RB, and MA. Supervision: GL, VB, AP, and SA. Critical discussion and final revision: RT and GL. All authors contributed to the article and approved the submitted version.

## Conflict of Interest

The authors declare that the research was conducted in the absence of any commercial or financial relationships that could be construed as a potential conflict of interest.

## Publisher’s Note

All claims expressed in this article are solely those of the authors and do not necessarily represent those of their affiliated organizations, or those of the publisher, the editors and the reviewers. Any product that may be evaluated in this article, or claim that may be made by its manufacturer, is not guaranteed or endorsed by the publisher.
