# Supplementary material for: The Role of Age on Beta-Amyloid1–42 Plasma Levels in Healthy Subjects
Source: Front Aging Neurosci. 2021 Aug 31;13:698571. doi: 10.3389/fnagi.2021.698571 (PMC8438760; doi:10.3389/fnagi.2021.698571)
Supplement: Supplementary file 1 [file Data_Sheet_1.docx]

### Assumption Tests on Linear Regression Models

**Overall Model (not age stratified): n = 275 subjects**


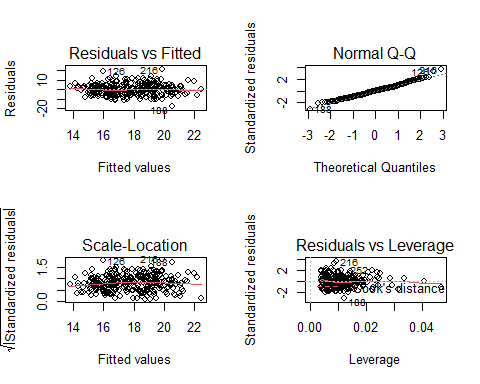

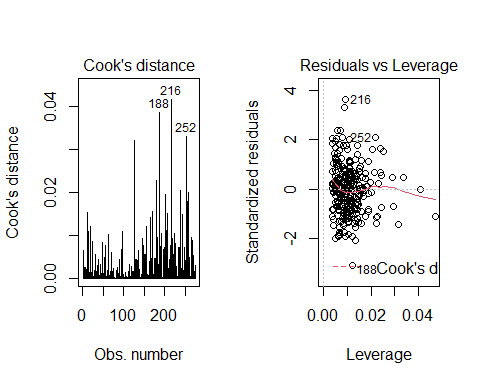


## [1] "Mean of residuals: 0.000000000000000225355774187571"

## [1] "Xs variances: "

## AGE AST ALT GGT
## 444.34723291 108.76345056 103.41425348 258.62739217
## BILIRUBINE_TOT Total_Cholesterol Triglycerides HDL
## 0.13587367 949.77443928 1964.14027700 136.14951559
## CREATININE GLUCOSE Tot_Protein TSH
## 0.09103327 339.57934970 0.31204380 0.63761924
## UREA WBC Erythrocytes PLATELETS
## 127.90710020 2.55488379 0.30838214 3240.00549436
## Hb Biomarker
## 2.43915727 35.67884737

## [1] "final model VIF: "

## AGE Total_Cholesterol
## 1.000034 1.000034

##
## Call:
## lm(formula = f, data = data)
##
## Coefficients:
## (Intercept) AGE Total_Cholesterol
## 8.49517 0.07613 0.02958
##
##
## ASSESSMENT OF THE LINEAR MODEL ASSUMPTIONS
## USING THE GLOBAL TEST ON 4 DEGREES-OF-FREEDOM:
## Level of Significance = 0.05
##
## Call:
## gvlma::gvlma(x = step.1, alphalevel = 0.05)
##
## Value p-value Decision
## Global Stat 11.88718 0.05821 Assumptions acceptable.
## Skewness 3.73270 0.05336 Assumptions acceptable.
## Kurtosis 2.45815 0.11692 Assumptions acceptable.
## Link Function 0.01407 0.90557 Assumptions acceptable.
## Heteroscedasticity 5.68226 0.01714 Assumptions NOT satisfied!^[[1]](#footnote-1)^

**0-35 Model: n = 93 patients.**


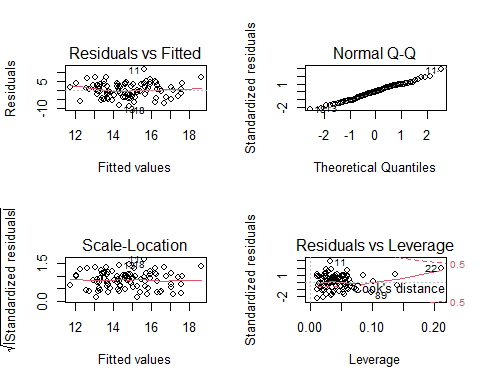

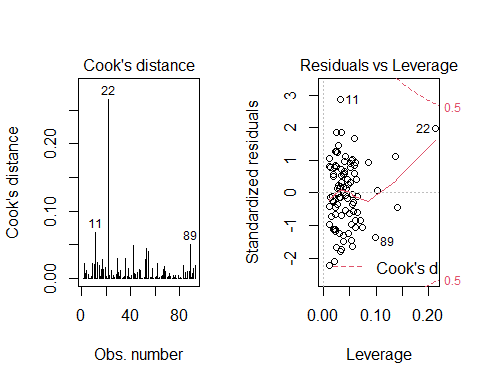


## [1] "Mean of residuals: -0.000000000000000129050369554253"

## [1] "Xs variances: "

## AGE AST ALT GGT
## 444.34723291 108.76345056 103.41425348 258.62739217
## BILIRUBINE_TOT Total_Cholesterol Triglycerides HDL
## 0.13587367 949.77443928 1964.14027700 136.14951559
## CREATININE GLUCOSE Tot_Protein TSH
## 0.09103327 339.57934970 0.31204380 0.63761924
## UREA WBC Erythrocytes PLATELETS
## 127.90710020 2.55488379 0.30838214 3240.00549436
## Hb Biomarker
## 2.43915727 35.67884737

## [1] "final model VIF: "

## CREATININE GLUCOSE AGE
## 1.035921 1.036274 1.002971

##
## Call:
## lm(formula = f, data = data)
##
## Coefficients:
## (Intercept) CREATININE GLUCOSE AGE
## 25.35498 2.75030 -0.07476 -0.23558
##
##
## ASSESSMENT OF THE LINEAR MODEL ASSUMPTIONS
## USING THE GLOBAL TEST ON 4 DEGREES-OF-FREEDOM:
## Level of Significance = 0.05
##
## Call:
## gvlma::gvlma(x = step.1, alphalevel = 0.05)
##
## Value p-value Decision
## Global Stat 7.392052 0.11656 Assumptions acceptable.
## Skewness 0.002012 0.96423 Assumptions acceptable.
## Kurtosis 0.308728 0.57846 Assumptions acceptable.
## Link Function 3.569728 0.05884 Assumptions acceptable.
## Heteroscedasticity 3.511584 0.06094 Assumptions acceptable.

**35-65 Model: n = 89 subjects.**


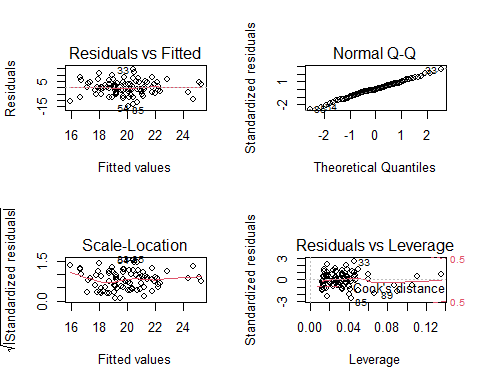

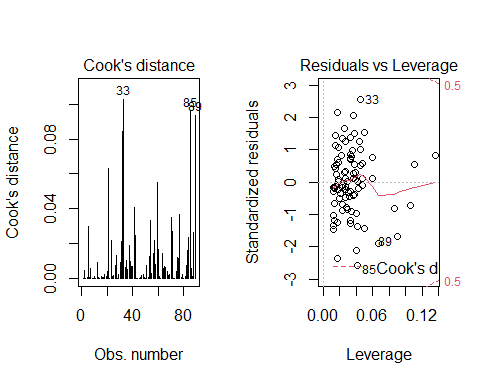


## [1] "Mean of residuals: 0.0000000000000000465841472762311"

## [1] "Xs variances: "

## AGE AST ALT GGT
## 444.34723291 108.76345056 103.41425348 258.62739217
## BILIRUBINE_TOT Total_Cholesterol Triglycerides HDL
## 0.13587367 949.77443928 1964.14027700 136.14951559
## CREATININE GLUCOSE Tot_Protein TSH
## 0.09103327 339.57934970 0.31204380 0.63761924
## UREA WBC Erythrocytes PLATELETS
## 127.90710020 2.55488379 0.30838214 3240.00549436
## Hb Biomarker
## 2.43915727 35.67884737

## [1] "final model VIF: "

## TSH AGE
## 1.025979 1.025979

##
## Call:
## lm(formula = f, data = data)
##
## Coefficients:
## (Intercept) TSH AGE
## 9.3655 2.3027 0.1416
##
##
## ASSESSMENT OF THE LINEAR MODEL ASSUMPTIONS
## USING THE GLOBAL TEST ON 4 DEGREES-OF-FREEDOM:
## Level of Significance = 0.05
##
## Call:
## gvlma::gvlma(x = step.1, alphalevel = 0.05)
##
## Value p-value Decision
## Global Stat 2.5299853 0.6393 Assumptions acceptable.
## Skewness 0.0882342 0.7664 Assumptions acceptable.
## Kurtosis 0.0001459 0.9904 Assumptions acceptable.
## Link Function 0.0096497 0.9217 Assumptions acceptable.
## Heteroscedasticity 2.4319555 0.1189 Assumptions acceptable.

**65+ Model: n = 93 patients.**


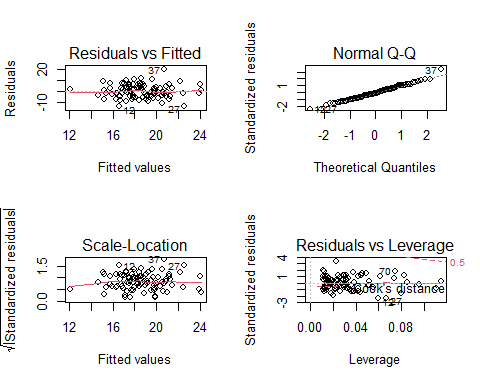

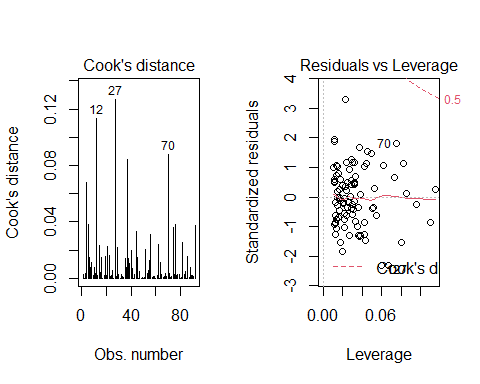


## [1] "Mean of residuals: -0.000000000000000179166765964474"

## [1] "Xs variances: "

## AGE AST ALT GGT
## 444.34723291 108.76345056 103.41425348 258.62739217
## BILIRUBINE_TOT Total_Cholesterol Triglycerides HDL
## 0.13587367 949.77443928 1964.14027700 136.14951559
## CREATININE GLUCOSE Tot_Protein TSH
## 0.09103327 339.57934970 0.31204380 0.63761924
## UREA WBC Erythrocytes PLATELETS
## 127.90710020 2.55488379 0.30838214 3240.00549436
## Hb Biomarker
## 2.43915727 35.67884737

## [1] "final model VIF: "

## Tot_Protein Total_Cholesterol
## 1.000413 1.000413

##
## Call:
## lm(formula = f, data = data)
##
## Coefficients:
## (Intercept) Tot_Protein Total_Cholesterol
## 35.39724 -3.39974 0.03668
##
##
## ASSESSMENT OF THE LINEAR MODEL ASSUMPTIONS
## USING THE GLOBAL TEST ON 4 DEGREES-OF-FREEDOM:
## Level of Significance = 0.05
##
## Call:
## gvlma::gvlma(x = step.1, alphalevel = 0.05)
##
## Value p-value Decision
## Global Stat 3.8191684 0.4310 Assumptions acceptable.
## Skewness 1.0404624 0.3077 Assumptions acceptable.
## Kurtosis 0.2965637 0.5860 Assumptions acceptable.
## Link Function 0.0009276 0.9757 Assumptions acceptable.
## Heteroscedasticity 2.4812147 0.1152 Assumptions acceptable.

1. Sandwich Estimator undertaken as well. No different estimates in terms of coefficients with respect to plain R base::lm method were obtained. [↑](#footnote-ref-1)
